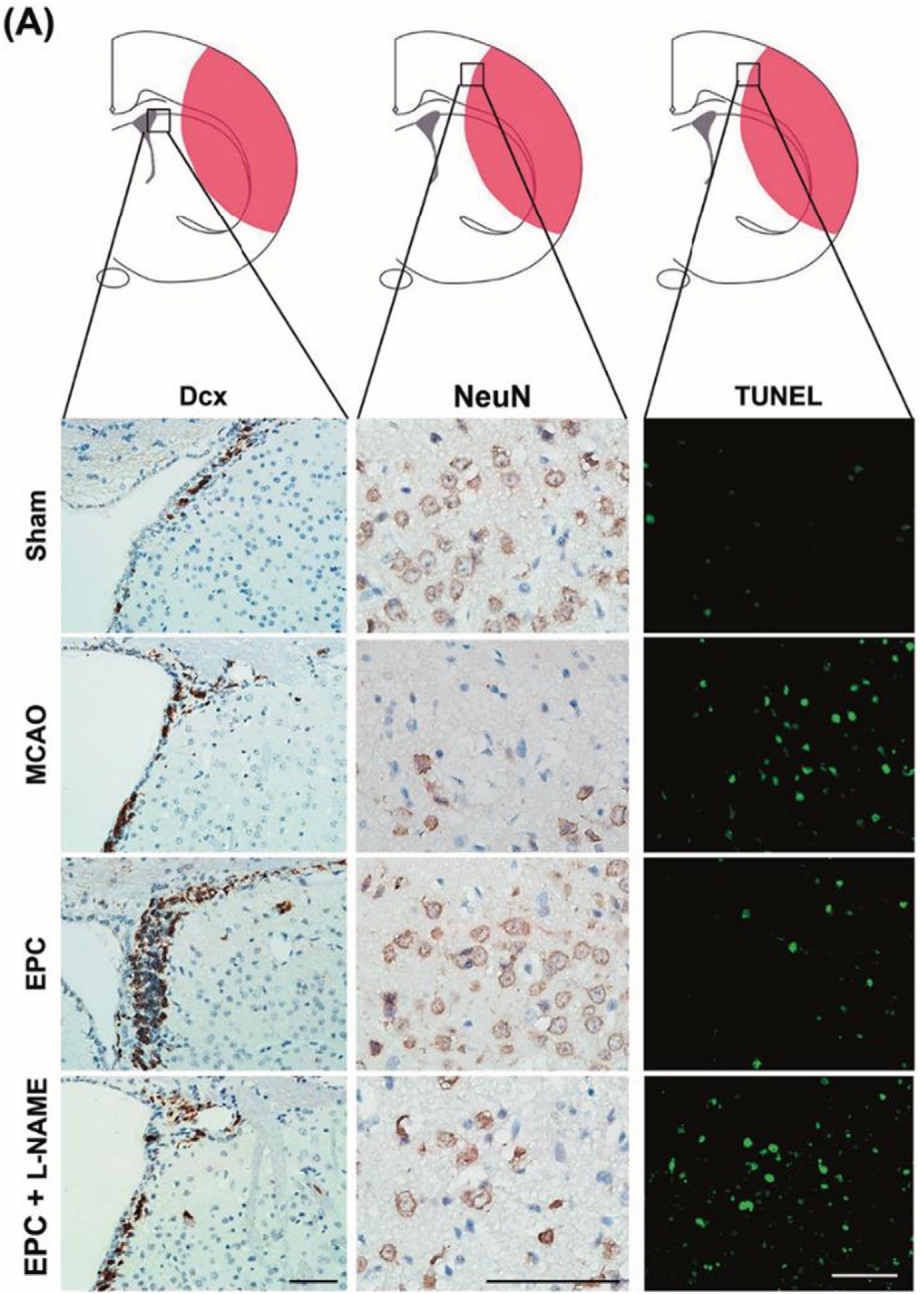# Correction to “Bone Marrow Endothelial Progenitor Cell Transplantation After Ischemic Stroke: An Investigation Into Its Possible Mechanism”

**DOI:** 10.1111/cns.70387

**Published:** 2025-04-09

Y. Y. Bai, X. G. Peng, L. S. Wang, Z. H. Li, Y. C. Wang, C. Q. Lu, J. Ding, P. C. Li, Z. Zhao, and S. H. Ju, “Bone Marrow Endothelial Progenitor Cell Transplantation After Ischemic Stroke: An Investigation Into Its Possible Mechanism,” *CNS Neuroscience & Therapeutics* 21, no. 11 (2015): 877–886. https://doi.org/10.1111/cns.12447.

In the original version of our article, there was an error in Figure 3A. Specifically, the immunohistochemical image of NeuN for the MCAO group was incorrect. The correct image is provided below. This correction will not affect the results and conclusion.

We apologize for this error.